# Long Noncoding RNA RAET1K Enhances CCNE1 Expression and Cell Cycle Arrest of Lung Adenocarcinoma Cell by Sponging miRNA-135a-5p

**DOI:** 10.3389/fgene.2019.01348

**Published:** 2020-01-17

**Authors:** Chang Zheng, Xuelian Li, Yangwu Ren, Zhihua Yin, Baosen Zhou

**Affiliations:** ^1^ Department of Clinical Epidemiology, First Affiliated Hospital of China Medical University, Shenyang, China; ^2^ Department of Epidemiology, School of Public Health, China Medical University, Shenyang, China; ^3^ Key Laboratory of Cancer Etiology and Intervention, University of Liaoning Province, Shenyang, China

**Keywords:** RAET1K, cell cycle, lung adenocarcinoma, long noncoding RNA, gene regulatory networks

## Abstract

Molecular dysregulation is believed to participate in the onset and progression of lung adenocarcinoma (LUAD). This study aimed to identify and evaluate the potential key long noncoding RNAs (lncRNAs) involved in the significant dysfunctional process of LUAD. We found that lncRNA retinoic acid early transcript 1K (RAET1K) was upregulated in tumor tissues and were correlated with a poor prognosis of patients with LUAD; further, for the first time, we detected the biological roles of RAET1K. Weighted gene correlation network and gene set enrichment analysis revealed that high RAET1K expression is related to cell cycle dysfunction through upregulated cyclin E1 (CCNE1) by targeting miR-135. The dual-luciferase reporter gene assay was performed to clarify the binding relationship between RAET1K and miR-135a-5p in transgenic A549 and H1299 cells. Real-time PCR and Western blot analyses showed that RAET1K overexpression and miR-135a-5p inhibition exerted a strong synergistic effect on CCNE1 expression, and cell cycle flow cytometry analysis was used to confirm the arrest of A549 and H1299 cells at the G1/S phase. The lncRNA RAET1K/miR-135a-5p axis might participate in the regulation of LUAD progression by influencing CCNE1 expression and the accumulation of cells arrested at the G1/S phase boundary.

## Introduction

The latest report released by the International Agency for Research on Cancer has stated that lung cancer (LC) remains the most common and deadly form of malignancy (Siegel et al., 2017; Bray et al., 2018). In general, surgery is the best option for treating patients with early stage disease because the five-year survival rate of pathological stage I non-small cell LC (NSCLC) after lobectomy is 45%–65% (Ettinger et al., 2015). However, approximately 70% of patients are diagnosed in the late stage of the disease; therefore, the five-year survival rate of these patients is only 16.38% (Ettinger et al., 2015). Lung adenocarcinoma (LUAD) is the most common type of NSCLC, accounting for approximately 40% of cases (Ferlay et al., 2010). Therefore, the focus of the present study was limited to the complex molecular mechanisms leading to the onset and poor prognosis of LUAD.

Dysregulation of the cell cycle result in increased cell proliferation, and the abnormal expression of cell cycle regulators can lead to tumor formation (Otto and Sicinski, 2017). Various chemotherapeutic agents have been developed to target the cell cycle (Ingham and Schwartz, 2017). For example, cisplatin is one of the most successful anticancer drugs used to nonspecifically block the cell cycle (Besse and Le Chevalier, 2012). By focusing on the complex gene networks that cause dysregulation of cell cycle regulators, a potential strategy for the treatment of LC could be developed.

Previous studies have reported that noncoding RNAs, such as long noncoding RNAs (lncRNAs) and microRNAs (miRNAs) are involved in cell cycle processes (Djebali et al., 2012). Furthermore, it has been widely reported that lncRNAs functioning as the competing endogenous RNAs (ceRNAs) could regulate cancer by sponging miRNAs (Salmena et al., 2011; Dong et al., 2018; Dong et al., 2019). Despite the rapid evolution of genomic technologies and analytical tools, the identification of novel lncRNA-related ceRNA networks affecting the cell cycle and ultimately influencing LUAD remains challenging. Therefore, the present study aimed to investigate lncRNA expression profiles of The Cancer Genome Atlas (TCGA) database *via* complex bioinformatics analysis to identify novel lncRNAs and related biological functions, which initially identified that lncRNA retinoic acid early transcript 1K (RAET1K) was significantly upregulated. Furthermore, we revealed that the upregulated expression of lncRNA RAET1K was correlated with poor prognosis in LUAD patients and facilitated cell cycle arrest at the G1 phase by functioning as a ceRNA to upregulate cyclin E1 (CCNE1).

## Material and Methods

### Data Sets and Preprocess

The RNA and miRNA sequence data of LUAD and corresponding clinical information were downloaded from the TCGA database (https://cancergenome.nih.gov). The study cohort consisted of 564 LUAD patients with level 3 Illumina HiSeq RNA sequencing (RNA-seq) data and 505 patients with level 3 miRNA sequencing (miRNA-seq) data. On the basis of the clinical traits of the patients, the samples were classified into two groups: early stage (stages I and II) and advanced stage (stages III and IV). The gene symbol and type were converted from transcript IDs of RNA-seq data with the use of Genome Reference Consortium Human Build 38 patch release 12 (GRCh38.p12) of the Ensembl genome browser (http://asia.ensembl.org/biomart). The DESeq2 package (Love et al., 2014) was used to normalize raw data sets and identify differentially expressed genes (DIFF-genes). The cutoff values were an absolute value of log2 fold change of ≥2 and an adjusted probability (*P*) value of ≤ 0.01.

### Construction of Co-Expression Networks

The R package for weighted correlation network analysis (WGCNA) was used to build co-expression networks (Langfelder and Horvath, 2008). Significant DIFF-genes were selected to generate co-expression networks for both the early and advanced stages of NSCLC. Briefly, a connection-weighted adjacency matrix of pair-wise genes was initially built according to unsupervised classifications. In accordance with the scale-independent topological criterion, the acceptable soft threshold value was set to 5 on the basis of a correlation coefficient threshold of 0.85 (Zhang and Horvath, 2005). Thereafter, a topological overlap matrix (TOM) was initially built on the adjacency matrix. The dynamic tree cutting method was performed to cluster DIFF-genes into modules with 30 as the minimum module sizes of the genes and 0.25 as the cluster merge height, respectively. Each module contained genes with similar expression patterns. The gray module consisted of a cluster of unclassified genes. After defining the modules, the module eigengene (ME) values were calculated for all genes in each module. The correlations between the ME values and the LUAD patient clinical traits were calculated (Langfelder and Horvath, 2007). Several significantly associated gene sets were chosen for functional enrichment analysis.

### Prognostic Analysis

Survival analysis was performed with SPSS Statistics for Windows, version 17.0. (SPSS, Inc., Chicago, IL, USA). On the basis of the gene expression value of the lower or upper quartile, samples were categorized into two groups: low-exp and high-exp. The hazard ratio (HR) and estimated 95% confidence interval (CI) were calculated using the Cox proportional hazard regression model. Kaplan-Meier curves were plotted to estimate the overall survival (OS), and the log rank test was used for univariate comparisons. A *P* value < 0.05 was considered statistically significant. Furthermore, a nomogram was generated using a multivariate Cox regression model to evaluate the potential prognostic signature of lncRNA RAET1K for OS of LUAD patients.

### Function Annotation and Gene Set Enrichment Analysis (GSEA)

Gene ontology (GO) enrichment analysis was performed to identify the biological processes (BPs) of the module. Relevant genes in the Database for Annotation, Visualization, and Integration Discovery (DAVID) were visualized using bubble plots. The DIFF-genes in specific modules were clustered into various Kyoto Encyclopedia of Genes and Genomes (KEGG) pathway ontologies using the ClueGO plug-in for the visualization of nonredundant biological terms for large clusters of genes in a functionally grouped network (Bindea et al., 2009). According to the gene expression level, GSEA was performed to identify the BPs and biological functions of hub genes clustered into the modules (Subramanian et al., 2005). For miRNAs, the miRcode (Jeggari et al., 2012) database was used to identify target genes and binding sites based on seed complementarity and evolutionary conservation of the seed region of the miRNAs.

### Cell Lines and Culture Conditions

Human LUAD A549 and H1299 cell lines were routinely cultured in a Roswell Park Memorial Institute 1640 medium (Gibco, Carlsbad, CA, USA) supplemented with 10% fetal bovine serum and 100 U/ml of penicillin/streptomycin (Beijing Solarbio Science & Technology Co., Ltd., Beijing, China) in an incubator (Thermo Fisher Scientific, Waltham, MA, USA) at 37°C under an atmosphere of 5% CO_2_/95% air, as previously described (Zheng et al., 2018).

### Cell Transfection

Cells were inoculated into the wells of a six-well plate before transfection. The RAET1K overexpression lentivirus and a negative control (NC) lentivirus were purchased from GenePharma Co., Ltd. (Shanghai, China). The cells in each well were transfected with 10^6^ lentiviruses. Four days later, the transfection efficiency was evaluated by determining the proportion of green fluorescent protein-positive cells. A medium supplemented with 2 μg/ml of puromycin was used to screen out the A549 and H1299 cells that were unsuccessfully transfected with the RAET1K and NC lentiviruses.

Cells were transiently transfected with a group of miR-135a-5p mimics and inhibitors (GenePharma Co., Ltd.) by using jetPRIME^®^ transfection reagent (Polyplus-transfection S.A., Illkirch-Graffenstaden, France), as previously described (Zheng et al., 2018). The cells were harvested at 24 h after transfection for further use.

### RNA Isolation and Real-Time Polymerase Chain Reaction (RT-PCR) Analysis

Total RNA was extracted using the NucleoSpin RNA Plus kit (TaKaRa Biotechnology [Dalian] Co., Ltd., Dalian, China) in accordance with the manufacturer's protocol. RNA was reverse-transcribed to complementary DNA (cDNA) using the PrimeScript RT Reagent Kit (TaKaRa Biotechnology [Dalian] Co., Ltd.). RT-PCR analysis was performed using SYBR Green Master Mixture reagent (Takara Bio, Inc., Kusatsu, Shiga, Japan) and an ABI 7500-Fast Real-Time PCR system (Applied Biosystems, Carlsbad, CA, USA). The cycling conditions for cDNA amplification are described elsewhere (Zheng et al., 2018). The fold change in relative gene expression was calculated using the 2^−∆∆^Ct method with glyceraldehyde 3-phosphate dehydrogenase (GAPDH) as an internal reference. The primers used for RT-PCR are listed in **Supplementary Table S1**.

### Western Blot Analysis

Total protein isolated from cells was sonicated in ice-cold radio immunoprecipitation assay lysis buffer (Pierce Biotechnology, Waltham, MA, USA). Denatured proteins were separated by sodium dodecyl sulfate polyacrylamide gel electrophoresis and then transferred to a polyvinylidene fluoride membrane (EMD Millipore Corporation, Billerica, MA, USA), which was blocked with Tris-buffered saline and 5% skim milk for 2 h. Samples were incubated with primary antibodies against the cyclin E1 (*CCNE1*) gene (catalog no. 20808; dilution, 1:1000; Cell Signaling Technology, Inc., Danvers, MA, USA) at 4°C overnight. After rinsing, the membrane was incubated with horseradish peroxidase-conjugated anti-rabbit secondary antibody (#7074; dilution, 1:1000; Cell Signaling Technology, Inc.). The protein bands were visualized using an enhanced chemiluminescence kit (Wanleibio Co., Ltd., Shenyang, China) and the ChemiDoc™ Touch Imaging System (Bio-Rad Laboratories, Hercules, CA, USA). The degree of gray intensity was determined using ImageJ software (https://imagej.nih.gov/ij/) and normalized to that of GAPDH (#2118; dilution, 1:5000; Cell Signaling Technology, Inc.).

### Flow Cytometry Analysis

The cells were fixed with ice-cold 70% ethanol overnight and then resuspended in staining solution included with the cell cycle detection kit (Nanjing KeyGen Biotech. Co. Ltd., Nanjing, China). After incubation for 1 h at 37°C in the dark, the stained cells were subsequently analyzed by flow cytometer fluorescence-activated cell sorting (FACS) using the BD FACSCalibur™ Cell Analyzer system (BD Biosciences, San Jose, CA, USA).

### Dual-Luciferase Reporter Assay

A fragment of the wild-type (WT) RAET1K 3'-untranslated region (RAET1K-3'UTR-wt) contained a binding site downstream of the luciferase reporter gene, whereas the mutant-type RAET1K (RAET1K-3'UTR-mut) contained mutated biding sites (GenePharma Co., Ltd.). A549 and H1299 cells were transfected in the wells of 24-well plates, cultured until attachment, and co-transfected with miR-135a-5p mimics, miR-135a-5p inhibitors or the miR-NC encoded by the luciferase plasmids (RAET1K-3'UTR-wt or RAET1K-3'UTR-mut). Luciferase gene expression was monitored using the Dual-Luciferase^®^ Reporter Assay System (Promega Corporation, Madison, WI, USA), as described previously (Zheng et al., 2018). The results of experiments performed in triplicate were normalized to Renilla luciferase activity values.

### Statistical Analysis

Data are presented as the mean ± standard deviation. All statistical analyses were performed using Prism 8.0 software (GraphPad Software, Inc., La Jolla, CA, USA). Student's *t*-test and one-way analysis of variance were used to analyze two groups and more than two groups, respectively. The Pearson's correlation coefficient was used to identify correlations. Analysis of each sample was performed in triplicate. *P* < 0.05 was considered statistically significant.

## Results

### Significant Genes and Clusters With Functions Related to LUAD

#### DIFF-Genes in Early and Advanced Stages of LUAD

**Figure 1:**
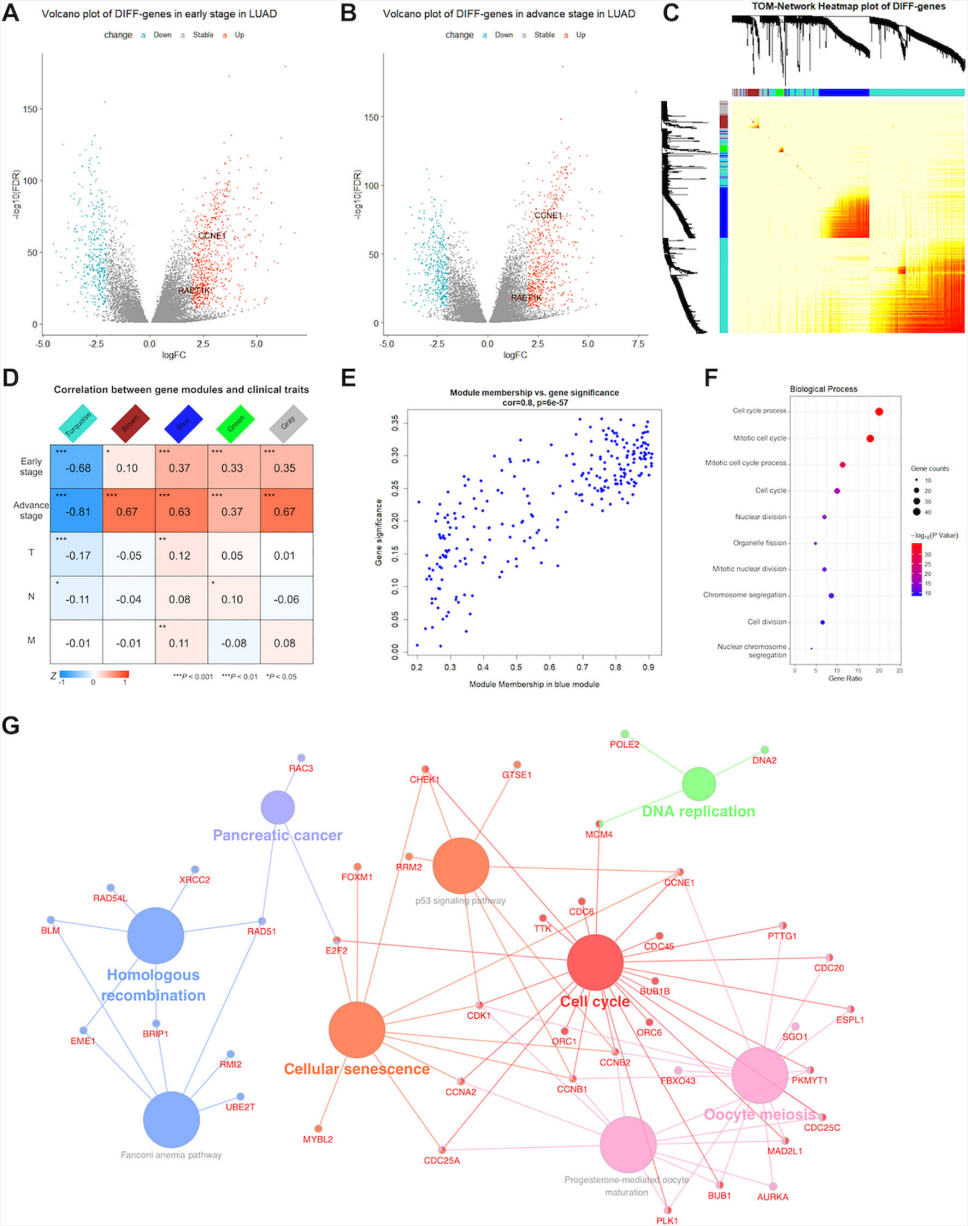
Detection of significant genes and their function related to lung adenocarcinoma (LUAD). Volcano plots showed fold change (FC) and *P*-values of differentially expressed genes in early **(A)** and advance **(B)** stage LUAD versus normal samples. Blue nodes present significantly down-regulated, and red nodes are up-regulated expressed genes. Grey nodes are not differentially expressed. RAET1K and CCNE1 expression are annotated. **(C)** In middle topological overlap matrix (TOM) heatmap, every row and column present one gene, light color presents low, while darker red presents higher weighted correlation. The dynamic tree cluster dendrogram of DIFF-genes are showed in the left and top, gray square indicates genes that are involved in any known module. **(D)** LUAD module-clinical feature relationships. The row matches a clinical trait (early stage, advance stage, T for tumor size, N for lymph node and M for metastasis) and the column matches a genes module. Correlation of module and clinical trait is showed in each cell. The darker the color is, the higher the degree of correlation is. Red presents positive, while blue presents negative correlation. **(E)** Scatterplot of gene significance and module membership in the blue module. Correlation coefficients and *P*-values are at the top. **(F)** Bubble plots showed top 10 terms of gene ontology (GO) enrichment analysis in biological process for blue module. The Y-axis correspond to the GO terms. The gene counts and -log (enrichment *P*-value) in every GO term were proportional to the area and color of the bubble, respectively. **(G)** Genes Kyoto Encyclopedia of Genes and Genomes (KEGG) enrichment analysis in blue module. The small size nodes in the network represent the genes enriched in the specific pathway, the big size nodes represent pathway term. The node colors correspond to the ClueGO-determined KEGG pathway clusters.

The LUAD database included 24,989 genes from 564 tissue samples, which included 59 adjacent noncancerous tissues, 395 early stage LUAD tissues (274 stage I and 121 stage II), and 110 advanced stage LUAD tissues (84 stage III and 26 stage IV). In total, 1,069 and 425 DIFF-genes were upregulated and downregulated in early stage LUAD (**Figure 1A**), respectively, whereas 888 and 516 were upregulated and downregulated in advanced stage LUAD, respectively (**Figure 1B**). In total, 991 DIFF-genes in both early and advanced stages were used to construct the weighted correlation network.

#### Construction of the Gene Co-Expression Network in LUAD

WGCNA was performed for 991 DIFF-genes. First, potential hub genes in each module were investigated to identify correlations with the clinical features of LUAD patients. The generalized TOM defined the relationships of each pair of DIFF-genes from the adjacency matrix. The hierarchical clustering tree method detected that four modules contained DIFF-genes that highly correlated with LUAD, as depicted in turquoise, brown, blue and green color (**Figure 1C**). In the middle of the TOM network, a heatmap of the independent genes in different modules was constructed. The genes clustered into the blue and turquoise modules were significantly co-expressed with each other.

The DIFF-genes in each module were spontaneously clustered according to the following clinical features: early stage, advanced stage, tumor size (T), lymph node involvement (N), and presence of metastasis (M). Module trait relationships were calculated by correlating the ME values with the clinical features (**Figure 1D**). There were no significantly positive modules related to early stage disease or other clinical traits. However, the genes in the blue and brown modules were significantly and positively correlated with advanced stage disease, whereas the genes in the blue module showed strong associations (correlation rate = 0.8, **Figure 1E**) and were chosen for subsequent analyses.

#### Functional Enrichment Analysis of Selected Modules

To describe the BPs and mechanisms of hub genes, the GO functional enrichment analysis of 203 DIFF-genes in the blue module were performed using DAVID as a reference. The top 10 BPs were visualized using a bubble plot (**Figure 1F**), which showed that most of the DIFF-genes were involved in the cell cycle (**Supplementary Table S2**). Furthermore, ClueGO was performed to enrich the KEGG pathways of the DIFF-genes in the blue module (**Figure 1G**). In total, 168 protein-coding RNAs in the blue module were grouped into six significant KEGG pathways (*P* ≤ 0.05). The red nodes contained 23 genes enriched in the cell cycle pathway (**Supplementary Table S3**).

### Function of RAET1K as a Key Gene in LUAD

#### Detection of Significant Genes in the Blue Module

According to GRCh38.p12, 12 lncRNAs and 191 mRNAs were assigned to the blue module. To further validate the hub genes and identify potential biomarkers for LUAD, Cox proportional hazard and Kaplan-Meier analyses of the genes in the blue module were performed. In total, 141 highly expressed hub genes were significantly associated with poor prognosis. Because there was only one lncRNA out of 141 significant genes in the blue module, and then we focused on this lncRNA RAET1K for further biological study.

#### RAET1K Is Highly Expressed in LUAD and Positively Correlated With the Prognosis of LUAD

**Figure 2:**
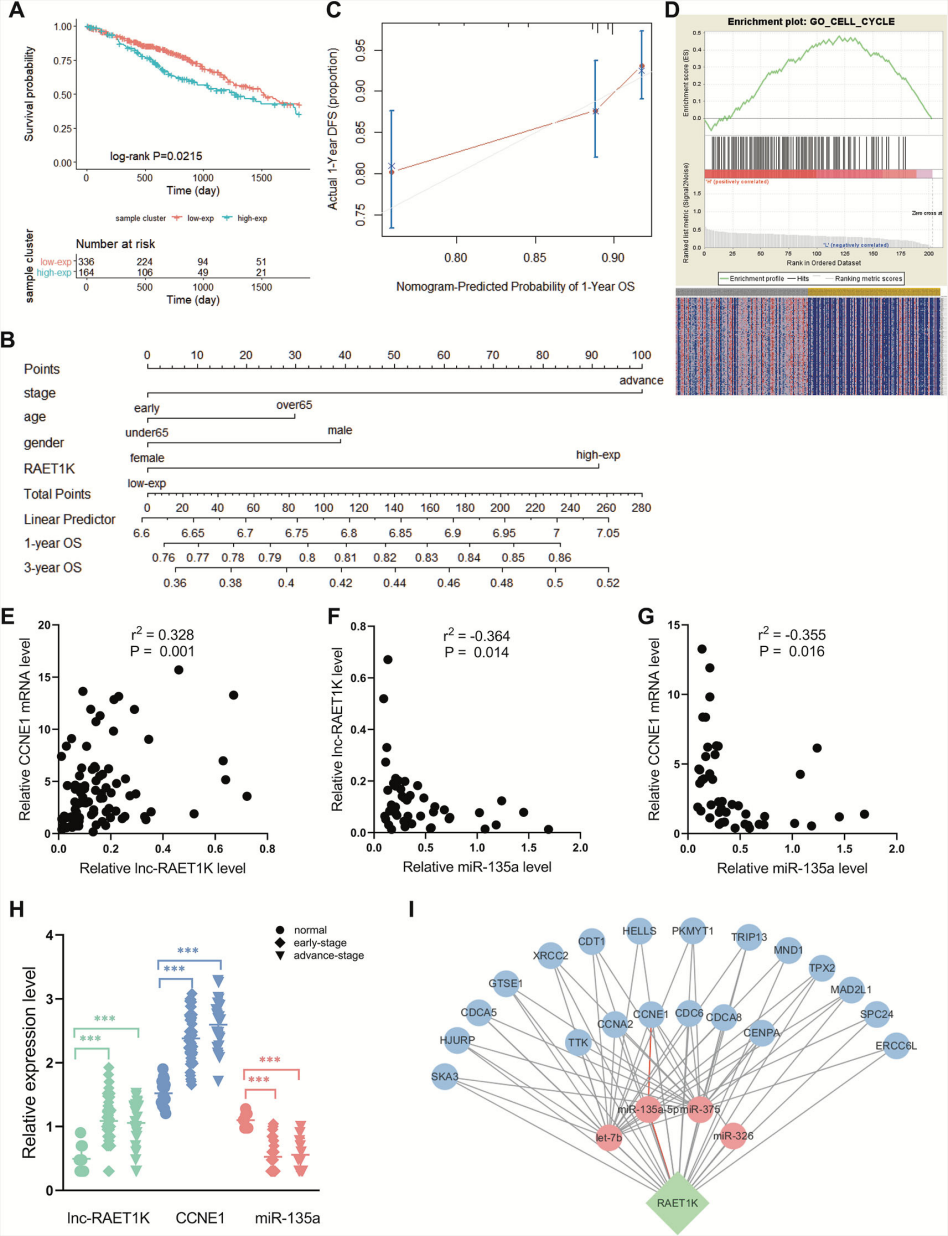
Identification of lncRNA RAET1K function and biological mechanism. **(A)** The Kaplan‐Meier curve of the risk score for the overall survival of RAET1K in lung adenocarcinoma (LUAD). The blue line presents the lower expression level group of RAET1K, and the red presents the higher ones. Gene enrichment plots showed gene set enrichment analysis (GSEA) between high- and low-expressed RAET1K. **(B)** The nomogram of clinical features and RAET1K expression level for predicting the 1- and 3-year survival with risk score. **(C)** calibration plot indicated this nomogram model had a predictive power for overall survival. **(D)** The upper enrichment plots contain value of the genes' enrichment scores and the corresponding barcode plot shows the genes position. In the bottom heatmap red represents Spearman correlations with higher expression level of RAET1K, blue represents Spearman correlations with lower expression level of RAET1K. Expression of RAET1K and CCNE1 expression level were positive related with each other **(E)**, while RAET1K **(F)** and CCNE1 **(G)** were negatively correlated with miR-135a. **(H)** RAET1K and CCNE1 expression were upregulated in both early and advance stage of LUAD, while miR-135a was downregulated, ***P < 0.001. **(I)** Construction of ceRNA network of lncRNA-miRNA-mRNA in blue module. The green node in diamond was lncRNA RAET1K, the blue circle nodes were mRNAs, and the pink circle nodes were miRNA. The line between nodes present their relation and the red lines shown RAET1K targeted miR-135a-5p and CCNE1.

RAET1K (HR = 1.428; 95% CI = 1.052–1.939; *P =* 0.022, **Figure 2A**) was the only lncRNA among the 141 hub genes that was significantly upregulated in tumor tissue compared with normal tissue (**Figure 2H**). Furthermore, a nomogram was constructed to predict 1- and 3-year survival rates in patients with LUAD by showing the risk score of clinical stage, age, sex, and RAET1K expression level (**Figure 2B**). The concordance index, which was evaluated using the calibration plot of this nomogram model, further supported the predictive prognostic signature of lncRNA RAET1K in LUAD OS (**Figure 2C**).

#### RAET1K May Regulate the Cell Cycle Phase in LUAD

To further explore the biological functions of RAET1K, GO enrichment for GSEA was performed. The LUAD samples with higher expression levels of RAET1K were enriched in genes correlated with cell cycle biological behavior. The GSEA results also indicated that among the genes in the blue module, lncRNA RAET1K expression was enriched in the cell cycle (**Figure 2D**).

#### The RAET1K/miR-135a-5p Axis May Influence the Cell Cycle *via* CCNE1 in LUAD Patients

lncRNAs can regulate mRNA expression *via* miRNA-mediated ceRNAs (Salmena et al., 2011). The expression of ceRNA transcripts that harbor the same miRNA binding sites should be parallel based on the ceRNA hypothesis. The interaction of the ceRNA network and RAET1K is described in **Figure 2I**, which was combined with the expressional correlation and target sites. Among the genes influencing OS, according to the Pearson's correlation coefficient, mRNAs that were positively correlated with RAET1K (r > 0.3 and *P* < 0.05, **Figure 2E**) and miRNAs that were negatively correlated with RAET1K and mRNAs (r < -0.3 and *P* < 0.05, **Figures 2F, G**) were selected, and then combined with the miRcode database, which was used to predict miRNA-interacting targets. As shown in **Figure 2I**, RAET1K may function as a sponge to absorb miR-135a-5p to modulate CCNE1 expression.

### The RAET1K/miR-135a-5p Axis Arrested LUAD Cells in the G1 Phase by Upregulating CCNE1

#### RAET1K Regulated CCNE1 by Sponging miR-135a-5p

**Figure 3:**
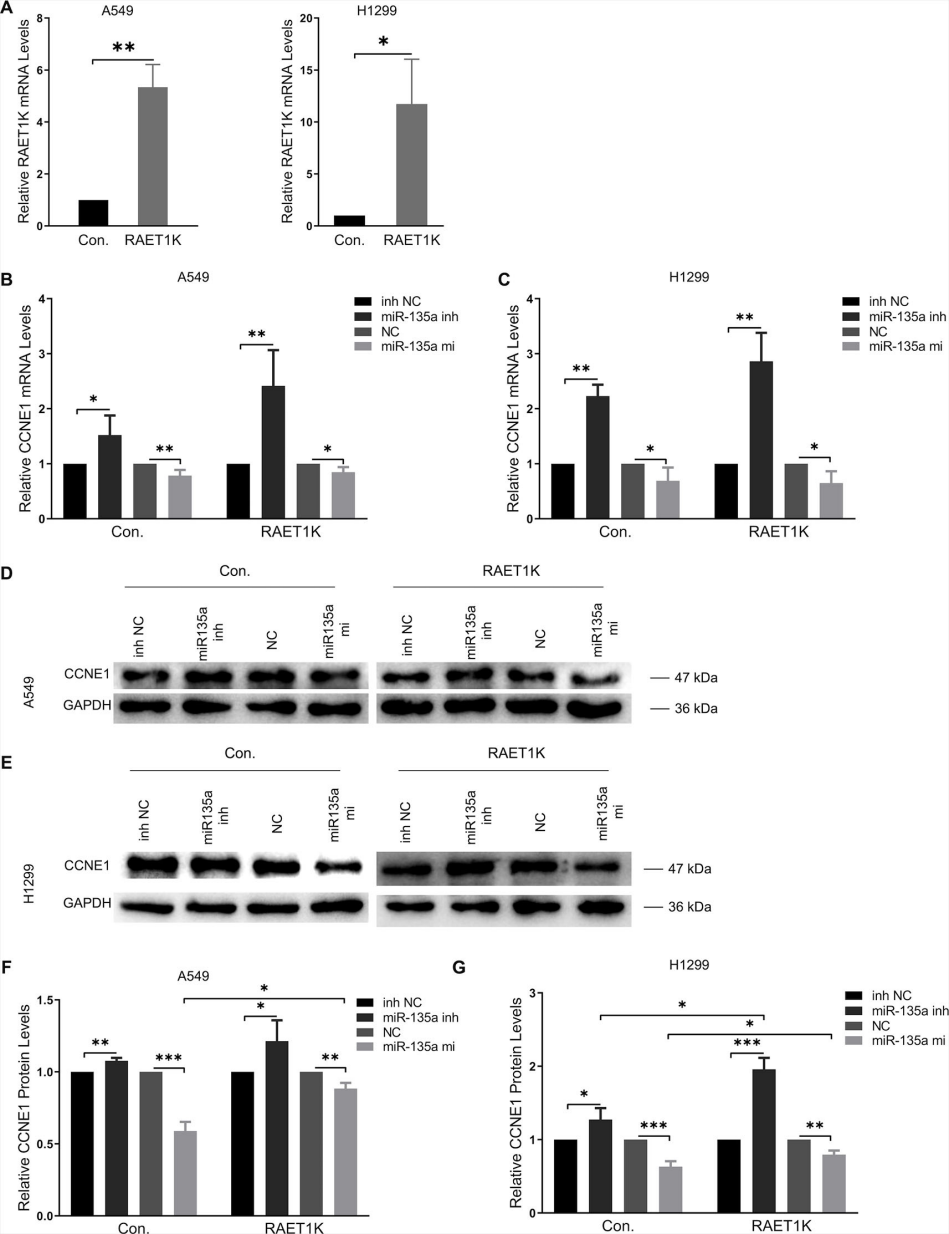
Overexpression RAET1K upregulated CCNE1 by sponging miR-135a-5p. **(A)** The interference efficiency of RAET1K overexpression lentivirus was detected by real-time PCR in A549 and H1299. Relative CCNE1 mRNA expression level after co-transfected with miR-135a-5p (or inhibitor) and RAET1K in A549 **(B)** and H1299 **(C)** cell lines, while the cyclin E1 protein levels was measured by Western blot in A549 **(D** and **F)** and H1299 **(E** and **G)**. Bands were quantitatively compared with relative negative control groups. Data are represented as means ± S.D. from three independent experiments, *P < 0.05, **P < 0.01, ***P < 0.001. Con., control; inh NC, miRNA-135a-5p inhibitor negative control; inh, inhibitor; NC, negative control; mi, mimics.

Subsequently, to investigate the validity and potential biological mechanisms of the effects of the RAET1K/miR-135a-5p axis on CCNE1 expression, *in vitro* experiments with A549 and H1299 cells were performed. The efficiency of RAET1K overexpression lentivirus interference was confirmed by RT–PCR (**Figure 3A**). To further investigate the synergistic effect of the RAET1K/miR-135a-5p axis on CCNE1 expression, A549 and H1299 cells were transfected with lentiviral vectors stably overexpressing RAET1K and an empty control (hereafter referred to as A549^RAET1K^, A549^Con^, H1299^RAET1K^, and H1299^Con^ cells, respectively). Thereafter, A549^RAET1K^, A549^Con^, H1299^RAET1K^, and H1299^Con^ cells were transfected with miR-135a-5p mimics, an inhibitor, an NC, or an NC inhibitor.

RT-PCR analyses of A549^Con^ and H1299^Con^ cells showed that miR-135a-5p inhibition resulted in a 1.5- and 2.2-fold increase, respectively, in CCNE1 mRNA expression relative to the NCs (**Figures 3B, C**, left panel). We observed that overexpression of RAET1K increased miR-135a-5p inhibition, as compared with NC (2.4- and 2.9-fold increases in A549^RAET1K^ and H1299^RAET1K^, respectively, **Figures 3B, C**, right panel).

Western blot analysis showed that cyclin E1 protein levels were similar (**Figures 3D–G**). We observed that A549^Con^ and A549^RAET1K^ cells transfected with miR-135a-5p mimics reduced cyclin E1 protein expression levels, whereas miR-135a-5p inhibitors had an opposite effect (**Figure 3D**). Consistently, cyclin E1 protein expression showed similar tendencies with higher fold changes in H1299^Con^ and H1299^RAET1K^ cells co-transfected with miR-135a-5p inhibitor compared with those with NC inhibitor (**Figure 3E**). Additionally, although miR-135a-5p mimics significantly decreased cyclin E1 protein expression, this change was salvaged by RAET1K overexpression, thereby indicating that the change in cyclin E1 protein expression in response to RAET1K and miR-135a-5p was due to posttranscriptional modulation in both A549 and H1299 cells. Considering these results, lncRNA RAET1K inhibited CCNE1 mRNA expression probably *via* the downregulation of miR-135a-5p expression.

#### RAET1K as a Target of miR-135a-5p

**Figure 4:**
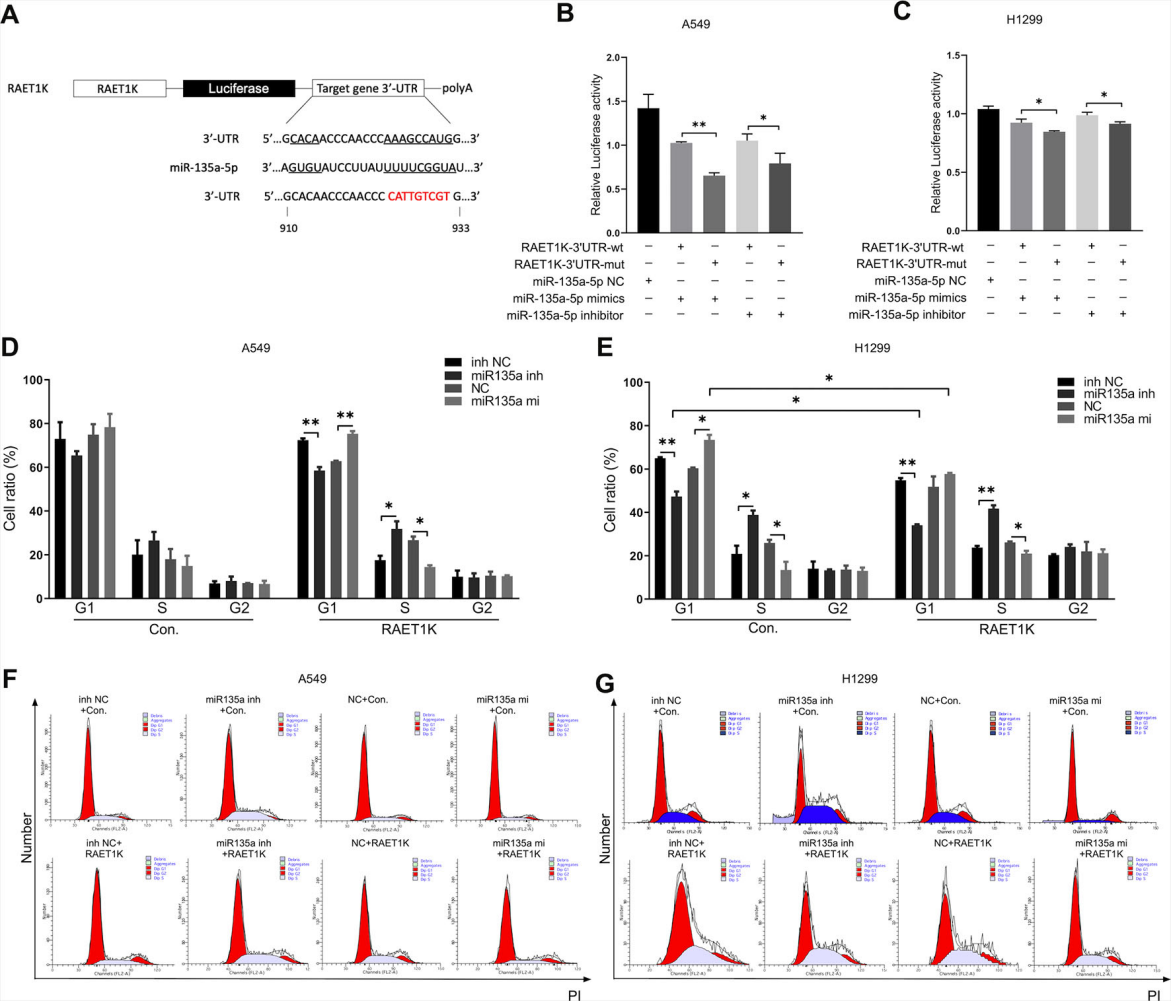
Upregulated RAET1K arrested G1 phase by targeted miR-135a-5p in lung adenocarcinoma (LUAD) cells. **(A)** Schematic representation of the putative binding target and modified sequence site of RAET1K for miR-135a-5p. Luciferase activity between RAET1K-3'UTR-wt/mut and miR-135a-5p detected by dual luciferase reporter assays in A549 **(B)** and H1299 **(C)**. The percentage of cell at different cell cycle phases were in the lower histograms in A549 **(D)** and H1299 **(E)**, while flow cytometry assay results showed cell cycle distribution by PI staining were presented in A549 **(F)** and H1299 **(G)**. Bands were quantitatively compared with relative negative control groups. Data are represented as means ± S.D. from three independent experiments, *P < 0.05, **P < 0.01. wt, wild type; mut, mutant type; inh NC, miRNA-135a-5p inhibitor negative control; inh, inhibitor; NC, negative control; mi, mimics; PI, propidium iodide.

The expression levels of miR-135a-5p and RAET1K were inversely correlated in LUAD tissues and cell lines. Bioinformatics analysis predicted that RAET1K was a potential target of miR-135a-5p. **Figure 4A** describes a putative interaction of RAET1K-3'UTR and modified RAET1K-3'UTR-mut with the miR-135a-5p binding sequence. The luciferase reporter assay was performed to validate the interactions between miR-135a-5p and RAET1K in A549 and H1299 cells. Relative luciferase activity was inhibited by co-transfection with the miR-135a-5p mimics and the luciferase reporters containing RAET1K-3'UTR. However, inhibition was relatively weak in the RAET1K-3'UTR-mut group (**Figures 4B, C**). Luciferase activity was enhanced with the use of the miR-135a-5p inhibitor (**Figures 4B, C**).

#### The RAET1K/miR-135a-5p Axis Arrested LUAD Cells in the G1 Phase

To determine whether the RAET1K/miR-135a-5p axis exerted synergistic effects on cell cycle progression, cell cycle distributions were investigated following the co-transfection of RAET1K and miR-135a-5p mimics or an inhibitor in A549 and H1299 cells. Although the proportions of A549^Con^ cells in the various cell cycle phases were not significantly altered by miR-135a-5p expression levels, a tendency for such alterations was observed (**Figure 4D**). In comparison with the NC group, transfection with the miR-135a-5p inhibitor decreased the number of A549^RAET1K^ cells in the G1 phase, whereas a larger proportion were observed in the S phase (**Figure 4D**).

Similar, yet significant, tendencies were observed in H1299 cells. As compared with the NC inhibitor group, the use of the miR-135a-5p inhibitor resulted in fewer H1299^Con^ and H1299^RAET1K^ cells arrested in the G1 phase than in the S phase (**Figure 4E**). In addition, lncRNA RAET1K overexpression enhanced the inhibition of cells arrested in the G1 phase. As compared with the NC group, transfection of H1299^Con^ cells with the miR-135a-5p mimics increased the number of cells accumulated in the G1 phase; however, RAET1K overexpression rescued this accumulation (**Figure 4E**). Moreover, histograms of the cell cycle were created (**Figures 4F, G**). The results showed that RAET1K overexpression with decreased miR-135a-5p could synergistically arrest the A549 and H1299 cells in the G1 phase and hinder cell cycle transformation from the G1 to S phase.

## Discussion

To identify significant lncRNAs in LUAD, comprehensive computational analysis of transgenic cells was performed. The results showed that lncRNA RAET1K regulated the expression of CCNE1 in LUAD and served as ceRNA to sponge miR-135a, whereas CCNE1 was targeted in cells arrested at the G1-S phase boundary. It is important to understand the pathological cell cycle process that is associated with the dysregulation of cell proliferation leading to cancer (Bertoli et al., 2013). The dynamic progression of the cell cycle consists of four sequential phases: S (chromosome replication), M (chromosome segregation), and G1 and G2 (gap), which are regulated by cyclin/cyclin-dependent kinases (Dai et al., 2018). In particular, cyclin E/Cdk2 interacts and forms complexes that promote G1 progression and G1/S transition (Sonntag et al., 2018). The amplification of cyclin E, which functions in cell cycle progression, inhibition of apoptosis, transcription, and replication, and DNA repair, has been observed in various types of cancer (Kanska et al., 2016; Vijayaraghavan et al., 2017). Furthermore, cyclin E1 can be modulated by multiple regulators, such as the transcription factors c-Myc, retinoblastoma, and E2F (Thurlings and de Bruin, 2016), as well as by miRNA-mediated inhibitors miR-15/16 (Yuan et al., 2019) and miR-424-5p(Jiang et al., 2019) at the transcriptional, posttranscriptional, and translational levels.

The rapid evolution of genomic technologies and analytical tools has improved the understanding of traditional simple gene mutations in cancer genomics. Furthermore elucidation of the complex networks of genomic alterations in LC has provided a basic understanding of the biological consequences and alterations of signal transduction pathways (Chin et al., 2011). A range of evidence suggests that diversity and complex molecular functions of lncRNAs may regulate epigenetic processes, particularly by acting as ceRNAs to sponge miRNAs. To identify novel LUAD-specific lncRNAs, differential analysis was performed during the early and advanced stages using normal tissues in the TCGA LUAD cohort. Different genes in both subsets were selected to facilitate the next step. The co-expression gene network was detected by WGCNA, which is a systematic biological method to identify synergistically altered gene clusters, candidate biomarkers, and therapeutic targets. According to the WGCNA results, DIFF-genes in the blue module were related to the LUAD clinical stage and were enriched in cell cycle-related functions. Cell cycle dysfunction in LUAD was consistent with our results. A recent study demonstrated that cell cycle-related genes, such as E2F1 (Chen et al., 2019), were enriched during the regulation of the cell cycle progression(Li et al., 2018; Qi et al., 2019). In the present study, we found that lncRNA RAET1K could promote cell cycle dysfunction, providing insight into the crosstalk regulatory mechanism between lncRNAs and coding genes. Interestingly, GSEA results also showed that some cell cyclin proteins and CDK family members were classified by the median of RAET1K expression level including PBK, KIF14, NEK2, CCNE1, CDC45, and DENPF, among others. In addition to the survival prediction of RAET1K, a Kaplan-Meier curve and a nomogram of integrating clinical traits were constructed. Indeed, RAET1K attracted our attention. Liang et al. (Sui et al., 2019) reported that RAET1K was predictive of the prognosis of LUAD patients in a TCGA cohort, which is consistent with our results; however, this was not further verified at the molecular level. To the best of our knowledge, no study has investigated the underlying molecular mechanism of RAET1K in patients with LUAD.

lncRNA RAET1K is a conversely processed transcript at 6q25.1 composed of four exons and is 1,883 bp in length. The key mechanism of lncRNA RAET1K as a ceRNA is to competitively combine the same miRNA with cross-regulated genes by sharing the miRNA response elements in the 3'-UTR of the target genes. We hypnotized that RAET1K functions as a ceRNA that influences CCNE1 expression and the cell cycle process *via* miR-135a-5p. The role of RAET1K in A549, H1299, and PC-9 cells was investigated to determine why PC-9 cells did not survive puromycin-selection of cells transfected with a lentivirus overexpressing RAET1K. As a possible explanation, the epidermal growth factor receptor gene might be mutated in PC-9 cells, whereas A549 and H1299 cell lines carried the WT phenotype. Therefore, the effects of miR-135a-5p and co-transfection of RAET1K/miR-135a-5p in A549 and H1299 cells were investigated. The results of the PC-9 cells transfected with miR-135a-5p are provided in the **Supplementary Figure S1**. In the A549 and H1299 cell lines, CCNE1 expression was silenced by increased miR-135a-5p, which also affected the cell cycle process. In contrast, the miR-135a-5p inhibitor had opposite effects. The results revealed that overexpression of RAET1K partially absorbed miR-135a-5p and enhanced the miR-135a-5p-mediated biological effects. The tumor suppressive function of miR-135a in LUAD has been consistently demonstrated in previous studies. For instance, miR-135a-5p promoted the progression of head and neck squamous cell carcinoma by targeting HOXA10 (Guo et al., 2018), the progression of thyroid carcinoma by VCAN (Zhao et al., 2017), and the progression of gastric cancer by KIFC1 (Zhang et al., 2016). Conversely, miR-135a was found to target SIAH1 to promote cell transformation in cervical cancer *via* the β-catenin pathway (Leung et al., 2014). Furthermore, Zhang et al. (2019) reported that miR-135a-5p promoted LC progression *via* modulating LOXL4 and blockage of LC cells arrested at the G1 phase. The reasons for these findings could be the differences in the samples used for *in vivo* (LC tissue) vs. *in vitro* (LC cell lines) studies. However, the results above were in agreement regarding the influence of the G1 phase of the cell cycle.

Furthermore, the results of this study indicated that co-transfection of A549^RAET1K^ and H1299^RAET1K^ cells with the miR-135a-5p inhibitor could act synergistically to reduce the expression level of CCNE1 and accumulate the proportion of cells arrested at the G1-S phase boundary, thereby suggesting the possible existence of an oncogenic RAET1K/miR-135a-5p axis. As predicted and verified by the bioinformatics algorithms and luciferase reporter assay, RAET1K and CCNE1 are potential targets of miR-135a-5p at the 7-mer-m8 site. The lncRNA RAET1K/miR-135a-5p axis might have a stronger synergistic effect on the regulation of cell cycle phase-dependent CCNE1 and transformation from the G1 to S phase. Here, the role of RAET1K as a putative oncogene in LUAD was revealed, suggesting that targeting the cyclin E1-CDK signaling provides a novel targeted therapeutic option for the treatment of LUAD. However, further investigations are required to verify the crucial molecules and signaling pathways involved in lncRNA RAET1K-mediated LUAD tumorigenesis.

## Conclusion

The major finding of this study was that RAET1K acted as a ceRNA and increased the expression of CCNE1 by directly competing with miRNA-135a-5p, which influenced the function of the cyclin E1 protein. Furthermore, the RAET1K/miR-135a-5p axis, which drives cell cycle progression, was arrested at the G1 phase in LUAD onset and progression. These findings are expected to be useful for the development of a novel biomarkers and pathways regulating the the cell cycle in LUAD.

## Data Availability Statement

The data that support the findings of this study are openly available in the Cancer Genome Atlas at (https://portal.gdc.cancer.gov).

## Author Contributions

Conceptualization, analysis and validation: CZ and XL. Software: YR and ZY. Writing: CZ. Funding acquisition: BZ and XL.

## Funding

This project was supported by the National Natural Science Foundation of China (No.81773524 and No.81502878).

## Conflict of Interest

The authors declare that the research was conducted in the absence of any commercial or financial relationships that could be construed as a potential conflict of interest.

## Supplementary Material

The Supplementary Material for this article can be found online at: https://www.frontiersin.org/articles/10.3389/fgene.2019.01348/full#supplementary-material
