# Supplementary material for: Long Noncoding RNA RAET1K Enhances CCNE1 Expression and Cell Cycle Arrest of Lung Adenocarcinoma Cell by Sponging miRNA-135a-5p
Source: Front Genet. 2020 Jan 17;10:1348. doi: 10.3389/fgene.2019.01348 (PMC6979007; doi:10.3389/fgene.2019.01348)
Supplement: Supplementary file 1 [file Table_1.docx]

Supplemental Table S1. Primer sequences used in the study.

| **Name** | **Sequence** |
| --- | --- |
| RAET1K-Forward primer | 5′-CACGGACACAGCAGAGGATCTTG-3′ |
| RAET1K-Reverse primer | 5′-CACGGTCACCTCCTTGTTCTTCTC-3′ |
| CCNE1-Forward primer | 5′-CCCATCATGCCGAGGGAG -3′ |
| CCNE1-Reverse primer | 5′-TATTGTCCCAAGGCTGGCTC -3′ |
| GAPDH -Forward primer | 5′-CAGGAGGCATTGCTGATGAT-3′ |
| GAPDH -Reverse primer | 5′-GAAGGCTGGGGCTCATTT-3′ |

Supplemental Table S2. Genes enrichment in top 10 biological process of GO.

|  | GO-ID | Description | Genes in GO term |
| --- | --- | --- | --- |
| 1 | GO:0022402 | cell cycle process | KIF23, KIFC1, XRCC2, PRC1, KNTC1, PKMYT1, TTK, AURKA, AURKB, PTTG1, FAM83D, KIF2C, CDCA8, OIP5, CDCA2, CDCA5, CCNA2, ASPM, CDCA3, CDC6, CDK1, KIF11, KIF15, TPX2, NUSAP1, MND1, ESPL1, PBK, UBE2C, RAD51, MAD2L1, SPAG5, ZWINT, BUB1B, NEK2, ANLN, CHEK1, CEP55, SPC24, SPC25, NCAPH, NCAPG, BUB1, FBXO43, SKA3, SKA1, HELLS, ERCC6L, TRIP13, EXO1, MKI67, DLGAP5, NUF2, CENPF, CDC20, BIRC5, NDC80, CENPE, CDC25C, RAD54L, CDC25A, CCNB1, CCNB2, PLK1 |
| 2 | GO:0000278 | mitotic cell cycle | KIF23, KIFC1, XRCC2, PRC1, KNTC1, PKMYT1, TTK, AURKA, AURKB, PTTG1, GTSE1, FAM83D, KIF2C, CCNE1, CDCA8, OIP5, CDCA2, CDCA5, CCNA2, ASPM, CDCA3, CDC6, CDK1, KIF11, KIF15, TPX2, NUSAP1, MND1, ESPL1, PBK, UBE2C, RAD51, MAD2L1, SPAG5, ZWINT, BUB1B, BLM, NEK2, ANLN, CHEK1, CEP55, SPC24, SPC25, NCAPH, NCAPG, BUB1, FBXO43, SKA3, SKA1, HELLS, ERCC6L, TRIP13, EXO1, MKI67, DLGAP5, NUF2, CENPF, CDC20, BIRC5, NDC80, CENPE, CDKN3, CDC25C, RAD54L, CDC25A, CCNB1, CCNB2, PLK1 |
| 3 | GO:1903047 | mitotic cell cycle process | KIF23, E2F2, KIFC1, CLSPN, XRCC2, PRC1, E2F7, E2F8, KNTC1, PKMYT1, TTK, AURKA, AURKB, PTTG1, GTSE1, CDT1, FAM83D, KIF2C, CCNE1, CDC45, CDCA8, OIP5, CDCA2, CDCA5, CCNA2, ASPM, CDCA3, CDC6, CDK1, KIF11, KIF15, TPX2, NUSAP1, MND1, ESPL1, PBK, UBE2C, ESCO2, RAD51, UHRF1, MAD2L1, SPAG5, ZWINT, BUB1B, BLM, NEK2, FOXM1, ANLN, CHEK1, CEP55, SPC24, SPC25, NCAPH, CENPA, NCAPG, HJURP, BUB1, FBXO43, SKA3, SKA1, HELLS, ERCC6L, TRIP13, EXO1, MKI67, DLGAP5, NUF2, CENPF, CDC20, BIRC5, NDC80, CENPE, CDKN3, CDC25C, RAD54L, CDC25A, CCNB1, CCNB2, PLK1 |
| 4 | GO:0007049 | cell cycle | KIF23, KIFC1, KNTC1, PKMYT1, AURKA, PTTG1, AURKB, FAM83D, KIF2C, CDCA8, OIP5, CDCA2, CDCA5, CCNA2, ASPM, CDCA3, CDC6, CDK1, KIF11, KIF15, TPX2, NUSAP1, ESPL1, PBK, UBE2C, MAD2L1, SPAG5, ZWINT, BUB1B, NEK2, ANLN, CEP55, SPC24, SPC25, NCAPH, NCAPG, BUB1, SKA3, SKA1, HELLS, ERCC6L, DLGAP5, NUF2, CENPF, BIRC5, NDC80, CENPE, CDC20, CDC25C, CDC25A, CCNB1, CCNB2, PLK1 |
| 5 | GO:0000280 | nuclear division | KIF23, KIFC1, KNTC1, PKMYT1, AURKA, PTTG1, AURKB, FAM83D, KIF2C, CDCA8, OIP5, CDCA2, CDCA5, CCNA2, ASPM, CDCA3, CDC6, CDK1, KIF11, KIF15, TPX2, NUSAP1, ESPL1, PBK, UBE2C, MAD2L1, SPAG5, ZWINT, BUB1B, NEK2, ANLN, CEP55, SPC24, SPC25, NCAPH, NCAPG, BUB1, SKA3, SKA1, HELLS, ERCC6L, DLGAP5, NUF2, CENPF, BIRC5, NDC80, CENPE, CDC20, CDC25C, CDC25A, CCNB1, CCNB2, PLK1 |
| 6 | GO:0048285 | organelle fission | KIF23, KIFC1, KNTC1, PKMYT1, AURKA, PTTG1, AURKB, FAM83D, KIF2C, CDCA8, OIP5, CDCA2, CDCA5, CCNA2, ASPM, CDCA3, CDC6, CDK1, KIF11, KIF15, TPX2, NUSAP1, ESPL1, PBK, UBE2C, MAD2L1, SPAG5, ZWINT, BUB1B, NEK2, ANLN, CEP55, SPC24, SPC25, NCAPH, NCAPG, BUB1, SKA3, SKA1, HELLS, ERCC6L, DLGAP5, NUF2, CENPF, BIRC5, NDC80, CENPE, CDC20, CDC25C, CDC25A, CCNB1, CCNB2, PLK1 |
| 7 | GO:0007067 | mitotic nuclear division | KIF23, KIFC1, PRC1, KNTC1, PKMYT1, TTK, AURKA, PTTG1, AURKB, GTSE1, FAM83D, KIF2C, CCNE1, CDCA8, OIP5, CDCA2, CDCA5, CCNA2, ASPM, CDCA3, CDC6, CDK1, KIF11, KIF15, TPX2, NUSAP1, ESPL1, PBK, UBE2C, MAD2L1, SPAG5, ZWINT, BUB1B, BLM, NEK2, ANLN, CHEK1, CEP55, SPC24, SPC25, NCAPH, NCAPG, CENPA, BUB1, SKA3, SKA1, HELLS, ERCC6L, DLGAP5, NUF2, CENPF, CDC20, BIRC5, NDC80, CENPE, CDKN3, CDC25C, CDC25A, CCNB1, CCNB2, PLK1 |
| 8 | GO:0007059 | chromosome segregation | KIF23, KIFC1, KNTC1, PKMYT1, AURKA, PTTG1, AURKB, FAM83D, KIF2C, CDCA8, OIP5, CDCA2, CDCA5, CCNA2, ASPM, CDCA3, CDC6, CDK1, KIF11, KIF15, TPX2, NUSAP1, ESPL1, PBK, UBE2C, MAD2L1, SPAG5, ZWINT, BUB1B, NEK2, ANLN, CEP55, SPC24, SPC25, NCAPH, NCAPG, BUB1, SKA3, SKA1, HELLS, ERCC6L, DLGAP5, NUF2, CENPF, BIRC5, NDC80, CENPE, CDC20, CDC25C, CDC25A, CCNB1, CCNB2, PLK1 |
| 9 | GO:0051301 | cell division | KIF23, KIFC1, XRCC2, PRC1, KNTC1, PKMYT1, TTK, AURKA, AURKB, PTTG1, GTSE1, FAM83D, KIF2C, CCNE1, CDCA8, OIP5, CDCA2, CDCA5, CCNA2, ASPM, CDCA3, CDC6, CDK1, KIF11, KIF15, TPX2, NUSAP1, MND1, ESPL1, PBK, UBE2C, RAD51, MAD2L1, SPAG5, ZWINT, BUB1B, BLM, NEK2, ANLN, CHEK1, CEP55, SPC24, SPC25, NCAPH, NCAPG, CENPA, BUB1, FBXO43, SKA3, SKA1, HELLS, ERCC6L, TRIP13, EXO1, MKI67, DLGAP5, NUF2, CENPF, CDC20, BIRC5, NDC80, CENPE, CDKN3, CDC25C, RAD54L, CDC25A, CCNB1, CCNB2, PLK1 |
| 10 | GO:0098813 | nuclear chromosome segregation | KIF23, KIFC1, PRC1, NEK2, KNTC1, ANLN, PTTG1, AURKB, CEP55, SPC24, FAM83D, CCNE1, SPC25, NCAPH, CDCA8, OIP5, NCAPG, BUB1, CDCA2, SKA3, SKA1, CDCA5, CCNA2, HELLS, ASPM, ERCC6L, CDCA3, CDK1, CDC6, KIF11, NUF2, CENPF, NUSAP1, CDC20, BIRC5, CENPE, NDC80, ESPL1, CDC25C, UBE2C, CDC25A, CCNB1, MAD2L1, CCNB2, SPAG5, PLK1, ZWINT, BUB1B |

Supplemental Table S3. Genes enrichment in KEGG pathways.

|  | KEGG-ID | Description | Genes in KEGG term |
| --- | --- | --- | --- |
| 1 | hsa04110 | cell cycle | E2F2, CDC6, CDK1, PKMYT1, TTK, CHEK1, ESPL1, CDC20, PTTG1, CDC25C, MCM4, CDC25A, CCNB1, CCNE1, CDC45, CCNB2, MAD2L1, PLK1, BUB1, BUB1B, CCNA2 |
| 2 | hsa04114 | oocyte meiosis | CDK1, PKMYT1, AURKA, CDC20, ESPL1, PTTG1, CDC25C, CCNB1, CCNE1, CCNB2, MAD2L1, PLK1, FBXO43, BUB1 |
| 3 | hsa04914 | progesterone-mediated oocyte maturation | CCNB1, CDK1, MAD2L1, CCNB2, PLK1, BUB1, PKMYT1, CDC25C, CCNA2, CDC25A |
| 4 | hsa04115 | p53 signaling pathway | CCNB1, CDK1, CCNE1, CCNB2, RRM2, CHEK1, GTSE1 |
| 5 | hsa03440 | homologous recombination | XRCC2, BLM, EME1, RAD54L, RAD51 |
| 6 | hsa03030 | DNA replication | DNA2, POLE2, MCM4 |
| 7 | hsa00240 | pyrimidine metabolism | POLE2, NME1, RRM2, TK1 |


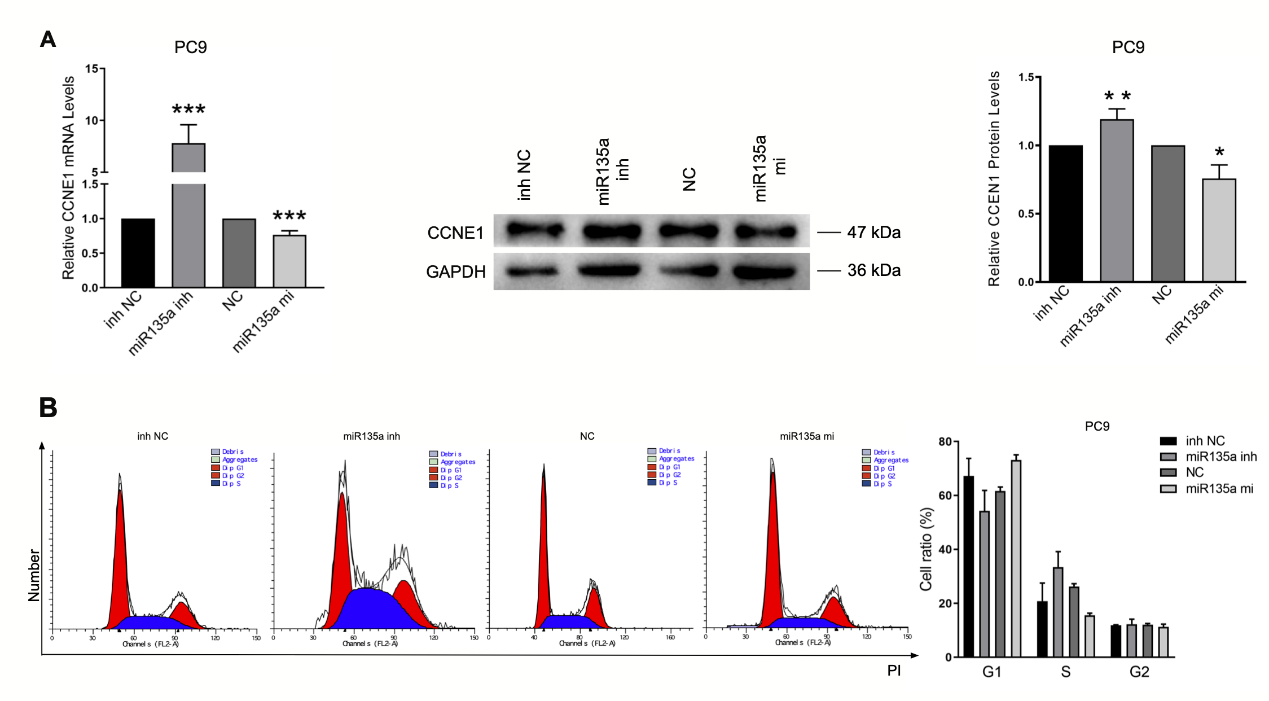


**Supplemental Figure S1.** Decreased miR-135a-5p arrested G1 phase by regulated expression of CCNE1 in PC9 cell lines. (A) The effect of miR-135a-5p on the mRNA expression level of CCNE1 was measured by real-time PCR while the cyclin E1 protein levels was measured by Western blot. (B) The flow cytometry assay results showed cell cycle distribution by propidium iodide staining were presented in the left pictures and percentage of cell at different cell cycle phases were in the right histograms. Bands were quantitatively compared with relative negative control groups. Data are represented as means ± S.D. from three independent experiments.

Abbreviations: inh NC, miRNA-135a-5p inhibitor negative control; inh, inhibitor; NC, negative control; mi, mimics.
